# The Experience of Being a Guide Dog Puppy Raiser Volunteer: A Longitudinal Qualitative Collective Case Study

**DOI:** 10.3390/ani5010001

**Published:** 2014-12-23

**Authors:** Anna Chur-Hansen, Lucy-Kate Werner, Clare E. McGuiness, Susan Hazel

**Affiliations:** 1 School of Psychology, University of Adelaide, South Australia, 5005, Australia; E-Mails: lucyw@live.com.au (L.-K.W.); clare.mcguiness@adelaide.edu.au (C.E.M.); 2 School of Animal Science, University of Adelaide, South Australia, 5005, Australia; E-Mail: susan.hazel@adelaide.edu.au

**Keywords:** guide dogs, health psychology, volunteering, human-animal studies, qualitative methods

## Abstract

**Simple Summary:**

Guide dogs are important service animals. They cannot be trained without the input of volunteer puppy raisers, who serve as custodians for the animals for around 12 months. To date very little research has considered the experience of being a guide dog puppy raiser, including the costs and benefits to psychological, physical and social health. In this study one litter of puppies and their raisers were followed from before the animal arrived until one year had passed. Overall, less positive experiences were reported than more negative ones. This has implications for the organisations that seek volunteers to raise service animals.

**Abstract:**

There are no published studies that consider the experiences of guide dog puppy raisers. As these people are volunteers, their continued willingness to participate in the training of dogs for assisting the vision impaired and blind is essential for the viability of guide dog schools around the world. Using a qualitative, longitudinal methodology, data were collected from nine guide dog puppy raisers at four time points: before receiving the puppy, one week, then three months after the puppy arrived, and 13 months after the puppy arrived (at which time all puppies had left the raisers). Participants reported more challenges than benefits in raising the puppies. Volunteering to be a guide dog puppy raiser may not be the pleasant experience that is anticipated when community members first offer their services. Understanding what it is like to be a puppy raiser and working towards ways in which to address problems is essential, given that, without volunteers to train and care for puppies, vision impaired and blind people would not have access to guide dogs.

## 1. Introduction

In Australia approximately 300,000 people are substantially vision impaired and around 20,000 are completely blind [1]. It is estimated that the overall incidence of vision impairment and blindness will increase in Australia, with the projected number by 2024 being 421,600 [1]. Of those who have vision impairment or blindness, some will apply for and qualify to receive a guide dog. The International Guide Dog Federation reports that there are 80 member schools around the world [2]. Whilst data on the number of guide dogs in Australia is unavailable, the Federation cites UK statistics, with 4500 guide dog owners and over 1200 puppies bred for training each year. Before someone can be matched with a suitable guide dog, the animal will have gone through extensive training. This training starts at around eight weeks of age, when the puppy joins its “foster family”, their raiser. Not all puppies that enter training will successfully graduate—some will be unsuitable for a number of reasons, which may include health (such as hip dysplasia) and temperament (such as anxiety or distractibility). Guide dog puppy raisers (also known as puppy educators and puppy walkers) are aware that they do not “own” the animal, but, rather, serve as a trainer and custodian for a period of approximately 12 months, after which time the animal undergoes further training for around three months, and then is placed with a vision-impaired person, to commence their working life.

There is a small body of literature on guide dogs. Researchers have considered how to best train the animals to become successful graduates [3,4], and these studies are, thus, within the purview of research on animal behaviour. Studies on the relationship between the guide dog and the vision-impaired person are few. In a study from South Africa by Wiggett-Barnard and Steel [5] six people with vision impairment who had a guide dog were interviewed. The authors concluded that living with a guide dog was life changing and offered both positive and negative experiences. For the participants in their study, the dogs improved mobility, provided companionship, and enhanced independence and personal growth. The dogs were often a source of pride for participants. On the other hand, living and working with a dog also meant having to change one’s lifestyle (with increased responsibility, time constraints, rearranging social engagements, and economic investment). The dogs were social magnets, but also sometimes repelled others, such as those who fear dogs. The participants experienced ignorance towards themselves and their dogs, with members of the public sometimes attempting to bar the person and their animal from entering premises. The dogs were well trained, but did not always manage to hold their attention to their tasks, becoming distracted.

The relationship between the puppy raiser and the guide dog in training appears to have been largely ignored in the research literature. This is surprising, given that if people in the community do not volunteer to raise the puppies and assist with training, the feasibility of guide dog schools would be compromised. Where researchers have included puppy raisers in their studies, this has tended to be as a source of information about the animal. For example, Valscchi *et al.* [6] investigated attachment between guide dogs in training and their raisers. However, the focus was on the dog’s attachment to the human, rather than the human raiser’s attachment to the animal. Koda [7,8] conducted a longitudinal study of the social play between 11 guide dog puppies and their raisers. Interactions were videotaped when the puppies were between the ages of two months to 11 or 12 months. The focus of the study was on the puppies and whether their play behaviours might be important in their development as guide dogs, as well as how to manage and discourage undesirable puppy behaviours. Gazzano *et al.* [9] have similarly included puppy raisers in research, but again, studied undesirable behaviours in guide dog puppies as observed by their raisers: the raisers themselves were not the focus of interest.

Whilst the research examining the effect of companion animals on human physical, psychological and social health is inconsistent [10], many studies suggest a positive relationship between companion animals and health [11]. This includes positive effects on physical, psychological, and social health [10]. It has also been argued that volunteering has positive effects on the physical, psychological, and social health of mid-life and older adults [12,13]. Therefore, it would be reasonable to posit that volunteering to be a guide dog puppy raiser would likely bring positive health benefits. However, to date, to the best of our knowledge, no previous published research has explored the impact raising and educating a guide dog puppy may have on human physical, psychological and social health. A search of the literature identified only one relevant unpublished doctoral dissertation [14], which surveyed 149 puppy raisers and concluded that the benefits outweighed the costs for the majority of respondents, and that altruism was the primary motivator for involvement in puppy raising. The current study aims to contribute to knowledge about the experience of being a guide dog puppy raiser, and poses the question “What is the impact of volunteering to raise a guide dog puppy on human physical, psychological and social health?”, using a longitudinal, qualitative collective case study research design.

## 2. Methods

### 2.1. Participants

Participants were volunteer guide dog puppy raisers and educators, recruited through an Australian guide dog school to raise and educate one litter of Labrador puppies. Puppies were the product of careful breeding to maximise the likelihood of successful training to become service animals. The raisers were volunteers recruited via advertisements in the media. To be accepted as a volunteer all were screened and selected based on an interview. Their property was carefully inspected for suitability to accommodate a puppy. All volunteers had to agree to attend initial and then subsequent regular training, take the dog with them everywhere (visiting, work, shopping, travel, including public transport), never leave the dog alone for more than two hours at any one time, and follow a prescribed training, diet and supervision routine.

Puppies’ allocated raisers were approached and asked to participate in the study. All nine raisers were women, and ranged in age from early thirties to late sixties. One participant had no previous experience living with a dog, but all others had previous experience with puppies and dogs in their households at some stage. All participants had lived with a companion animal in the past, and one was living with a dog at the time of the study, with one household incorporating chickens, one a rabbit, and three including cats. One participant was living alone at the time of the interviews; all others were living with a partner and/or children. Participants were interviewed in their homes. The University of Adelaide Human Research Ethics Committee approved the study. All participants provided informed consent.

### 2.2. Procedure

Participants were interviewed at four time points: prior to receiving the puppy (Time 1), within one week of receiving the puppy (Time 2), after 12 weeks from the arrival of the puppy (Time 3), and at 13 months after the puppy first arrived (Time 4). The second author conducted the first three interviews; the third author conducted the final interview. All interviews were audiotape recorded with permission. Both interviewers maintained an audit trail.

In-depth open-ended interviews lasting between 10 and 80 minutes were conducted. The average length of interviews was around 30 minutes. Participants were encouraged to speak freely about their thoughts and experiences as puppy raisers. Minimal prompts were made, so that participants could express what they felt was important. Where the participant was with a family member during the interview (which was the case for four of the participants), family members’ contributions were also included in analyses. However, the guide dog puppy raiser was the focus for all interviews. At the first interview the opening prompt was: “Can you tell me how you feel about becoming a guide dog raiser?” and “Do you think that being a guide dog raiser will be good for your health?” Further prompts included: “Can you talk to me about what you think it will be like to be a guide dog raiser?”, “What do you think your life will be like as a guide dog raiser?”, and “What are your motivations for doing this?” For the second, third and fourth interviews the following direct prompts were included: “Can you tell me about your puppy?”; “How has having the puppy changed your life?” and “Can you talk to me about the ways having the puppy has influenced your health?”

All interviews were transcribed verbatim. The first author read and re-read the transcripts and the audit trails. A substantial amount of data was produced, with 32 interviews (one participant was unavailable at Time 1, two participants did not participate in the Time 3 interview due to relinquishment of the puppy, and one participant was unavailable at Time 4). To manage the data, nine case studies were constructed by the first author, and then checked back to the original data by the fourth author. The case studies were then collapsed to provide a summary of the main findings across the four time points. To ensure trustworthiness and an accurate representation of the data, all four authors checked these summaries against the raw data. The methodology used followed the procedures outlined in Thomas [15] for collective case study designs.

## 3. Results and Discussion

### 3.1. Interview Time 1 (before the Puppy Arrived). N = 8 Participants

All participants expressed positive sentiments about the impending arrival, and several expressed excitement, and also nervous excitement. There was anticipation that the participant would receive psychological, physical and social benefits from the puppy, with expectations that the puppy would be calming, give the participant a sense of purpose, provide opportunities for the participant to learn new skills, increase family cohesion, allow spouses to function as a team, increase exercise through walking, and increase social interaction through activities related to the puppy. Sentiments of altruism were expressed, with a desire to help others, do something for someone else, and to do something through volunteer work that benefits the community. Additionally expressed were the perceived advantages of a short-term commitment to the animal, such as not having to face its death. Friends and work colleagues were reported to be supportive, although one partner was not keen on the puppy, but had agreed. The exercise was deemed worthwhile, and potentially interesting and rewarding, and several participants indicated that they expected this to be the first of subsequent puppy raising efforts. It was anticipated that any problems could be “nipped in the bud”. Participants were clear that they understood and accepted that the puppy would be leaving them at some stage.

There were concerns raised about the impending arrival. These included worry that the task would be substantial and require effort, that there would be rules and restrictions, some concern that relinquishment might be difficult, and concern about how existing animals would cope with the new addition. Anxiety was expressed about the success of the puppy and how this would reflect on the participant; concern was raised that if the puppy failed, someone would not get a guide dog. Thus, there was a sense of responsibility for participants that the puppy must succeed. Concerns were also expressed about the puppy’s welfare whilst in the participant’s care. Parallels were drawn with being responsible for the care of a baby, and the puppy was referred to as a baby. Specific concerns for participants included digging, dog hair, how existing companion animals would fare, and loss of sleep for the participant and others in the household. One participant discussed the need for support from the organization, expressing surprise that there had not been more training before the puppy’s arrival.

### 3.2. Interview Time 2 (after One Week from the Puppy’s Arrival). N = 9 Participants

The puppy was variously described as beautiful, cute, gorgeous, enjoyable, clever and learning quickly. The puppy was seen to have personality, acting as a support, soothing to brush, adding structure to the participant’s day, and teaching lessons to the household’s children. Children were reported to like, enjoy, and love the puppy, and families were working as a team together with the puppy.

However, overall, the feedback after one week with the puppy was overwhelmingly negative. The bad outweighed the good. Only one participant stated that there were no problems and no changes to their life, and notably, this puppy was surrendered soon after the interview. All other eight participants struggled with stress and “trying times”. One participant had already returned the puppy at the time of the interview, feeling bad, but saying that it was “a load off her mind”.

For participants, having to be with the puppy all the time and leaving it for no more than two hours was onerous. Vigilance was needed all the time, worrying and watching constantly. Participants reported toileting and cleanliness to be major issues, with urine and faeces in the house: one participant complained that the puppy had defecated on her. Sleep was disrupted, with the puppy crying for its littermates, the toilet, and because of teething. Clothes, shoes, hands and feet, bags, curtains, slippers, children’s and other dog’s toys, the phone cord, chairs, an electricity cable and a washing machine door were chewed, with one husband used as a “chew toy”. One participant’s infant child’s teddy and pacifier were being taken from the child by the puppy and chewed. Sleep deprivation was cited as detrimental to health, and also to family relationships, with members snappy, angry, grumpy and on edge. One participant was run down and sick, taking four days off work, with her children missing days from school because of interrupted sleep. The puppy was repeatedly compared to a child, with one participant claiming that it was more exhausting than being a single parent with a toddler.

One participant, who expressed a need for “space”, found the puppy demanding and clingy. For eight participants, the intensity of the puppy’s demands was unexpected. Puppies tested boundaries constantly, “always into mischief”, boisterous and excitable, displaying irritating sniffing, “crazy” and “mad” behaviour, particularly at meal-times, and venturing into the kitchen area where guide dogs in training are not allowed. Rules were harder to enforce than had been anticipated, and the rules and the puppy’s presence were restricting. Several participants confided that they had relaxed the rules in order to manage. It should be noted that at this stage the puppies were not vaccinated and thus could not be taken out: several participants commented on looking forward to being able to being able to leave the house with the puppy.

Being a puppy raiser was considered to be “really hard work”, and it had put one participant “off puppies forever”. Another stated that she would not have done this if she had known how hard it would be. Having the puppy was “bad for your health”, with no time to relax. It was harder than participants thought, and for one participant, life seemed to be at a standstill. Another had not done her hair for four days. It was a “shock to the system”, and a chore. Whilst one rabbit was not concerned about the new addition to the family, a cat and a dog were “unhappy” and the puppy was jealous of the existing dog and growling at her, which the participant found “frightening”. Empathy was expressed for the puppy: one participant felt that it was harsh to deprive a puppy of “doggie rights”, and another pondered that the puppy was probably very stressed too.

Participants indicated that they did not have enough information from the organization about normal puppy behaviour, and whether they were doing the right things. Additionally expressed was a need for praise and reassurance from the organization, which had not been given.

### 3.3. Interview Time 3 (Three Months after the Arrival of the Puppy). N = 7 Participants

By this time three puppies had been relinquished: one participant who had returned the puppy was interviewed. The puppy had killed a pet chicken, was constantly pulling the pacifier from the baby’s mouth and stealing the baby’s bottle for the milk. The participant was sad but relieved: the puppy had helped to get her out of the house and meet people, but her stress levels were “sky high”. The puppy was, for her, like caring for a toddler: she thought she might try being a puppy raiser again when her children were older.

For the remaining six puppy raisers feedback was generally negative. Some positives were reported however. The puppy was seen to be part of the family, and could be “quite sweet”, affectionate and comforting, with attachment expressed by several, but not all, participants: “we are starting to like her a great deal”. Children in the family were benefitting from the experience, and families were working together and couples feeling united in their task, leading to the perception of improved relationships. Laughing over the puppy’s funny antics was seen to be good for health. One participant described that the emotional, physical and social health of the whole family had been enhanced. Members of the public stopped and chatted about the puppy, intrigued about the process, making one participant feel that she was doing something valuable. A vision impaired person who thanked a participant for being a puppy raiser, as having the guide dog had changed his life, “refocused” them. Participants, who were all struggling, kept telling themselves that they were doing a good thing for someone, in order to persevere.

All participants were highly stressed. One participant wondered if they were “insane” to have done this. Another declared that she “hated” the puppy and had shed more tears over the experience than anything else in her life, describing it as stressful, draining and awful, “like a nightmare”. A further participant explained that they “were hating” the puppy by the end of each day. One stated that she was “still finding it extremely difficult, the whole process. Much more harder than what I ever imagined.” All participants gave variations of this statement, surprised at how challenging the experience was. Participants were exhausted and tired. One was saddened that there had been no positives to having the puppy. Puppies were described as demanding, dominating, attention seeking, over-excited, naughty and difficult, like an overtired child, biting, nipping, chewing, barking, mouthing and sniffing. Puppies were frustrated by the rules, angry and bored by restrictions and requiring intense supervision. One puppy was thought to goad the participant into “getting a response”, another described the puppy as clever and perceptive in knowing when it had “beaten her down”, yet another described it as a “contest” of wills. Participants compared the puppy to a baby, a grandchild and a teenager with selective hearing.

There were no health benefits reported. One participant had to go to the chiropractor: “I’m so sore and hurting everywhere. Because I have to walk her with my left hand, which is not strong, and she’s too strong for me. And we hadn’t mastered, and had enough training sessions, on walking. All my muscles were stretched and inflamed”. One participant and her mother had done a lot more walking, up to two hours in a day, and had thought that would be good for their fitness, but the participant felt she had in fact lost physical strength from managing the puppy, and was essentially exhausted. One participant was not sleeping, and consequently was nervous all the time and unable to concentrate at work.

In public outings with the puppy, participants were highly stressed. One puppy’s behaviour was “diabolical to very poor” in the supermarket. Whilst some members of the public were supportive and kind, their “humanity touching”, others were found to be abusive regarding training protocols and insistent on patting the puppy despite being asked not to. Puppies were “magnets” for people, yet walking the puppy through the city was described as “awful”, as people often did not get out of the way.

Socially, all participants were restricted, turning down invitations, the puppy “ruling their lives”. Some had had arguments with family and friends about the puppy, its behaviour and how they were managing the puppy. One participant could no longer cope with being “confined”, and had asked the organization for four days emergency respite from the puppy, so she and her husband could have a break to go to a party and the cinema. Family dynamics were adversely impacted when the puppy favoured one person over others: one couple found they had less time together as the puppy dominated the husband: he found it suffocating and needed “space”. However, another participant discussed the benefits of working as a team in the family, and that if it were only one person, then “neither would enjoy the experience”.

Participants expressed frustration and anger that there was not more support provided to them. They explained they needed training, information and counseling, role models and a support group to be a puppy raiser, as well as regular meetings. The organization was reported to be unsupportive, telling the participants “he’s a puppy”, when they sought help. One participant felt bad for the puppy, and stated that any failure would be the fault of the organization. One participant explained that she would be pleased to mentor anyone contemplating being a puppy raiser, and that she would be very honest.

Failure was on all participants’ minds, with disappointment expressed that the time and effort was not working to train the puppy. One participant was feeling “like a failure” and worried that the puppy would not “make it”. Another puppy, who was involved in an accident, left the participant disappointed, disheartened, and filled with uncertainty, waiting and anxious that perhaps the puppy would not become a guide dog now. The responsibility for participants was consuming: “we could do something wrong, which could ruin his little life”.

### 3.4. Interview Time 4 (13 Months after the Arrival of the Puppy). N = 8 Participants

Two of the participants who had relinquished the puppy by Time 3 were more positive about the puppy than others at this interview. One of these participants felt that the family had formed an attachment with the puppy, and that the puppy had impacted the family in positive and negative ways. It was not traumatic to relinquish her, as it was always understood she was “on loan” and therefore there was always a distance, but she had been missed. The participant who had relinquished the puppy after one week explained that full-time work had made it impossible to keep the puppy, but that they had bonded with the puppy. This participant felt there were no physical or mental health benefits or detriments to having the puppy. It seemed like “something was missing” when they had to relinquish the puppy, but there were no problems in doing so. She stated that she would recommend to others to be a guide dog puppy raiser, and would do it again if she were at home full-time. Working full-time was also problematic for the third participant. She felt that being a puppy raiser was definitely a worthwhile contribution to society, and would recommend it to the “right people”: the right people would need to know “how much work it is”. For this participant the bad memories of the experience outweighed the good ones. “A huge weight” was lifted from her shoulders, when the puppy left, although a bond between them had definitely formed, and the participant was teary when she saw the puppy being driven away in a van. The participant felt that she had let the dog down by returning her, but had found it very hard to be strict, with continuous responsibility and rules. This participant said that one of her motivations had been to get fit by walking the puppy, but that she suffered a number of health problems after the puppy’s arrival, and whilst not sure if all of these could be attributed to the puppy, she believed her health problems were exacerbated by the stress of it all.

For five of the six participants who had the puppy at Time 3, the reflections about the experience were generally negative. One was very positive: her puppy had been withdrawn from the program, to her “dismay”. The family had considered keeping him, but decided against it. This participant subsequently volunteered to foster another guide dog puppy, the only participant of the nine to do so, as well as the only one to unreservedly say she would recommend the experience to others. This participant believed that the experience had improved the family’s mental, physical and social health, with a heightened awareness of their place in society and their responsibilities to others. The family’s walking had increased, having to think about the puppy’s diet had made them think more about their own, the family was closer because they had all worked towards a goal, and they had made friendships with other puppy raisers. Puppies were seen as “visiting family members”.

Five of the participants who had the puppy at Time 3 reflected at this interview that the experience was confusing, frustrating, a struggle, heartbreaking, “such a let down” and “a culture shock”. All of the negative reflections made at Time 3 were reiterated, with only one participant feeling that the situation improved as the puppy got older. Not all participants had retained the puppy for twelve months: one participant was relieved when it was discovered that the puppy had hip dysplasia, as “it ended it for both of us”. The participant felt sadness that the puppy would not become a guide dog but relief that he was gone. Another, who did keep the puppy for one year, regretted not returning him earlier, deciding to struggle through, saying that “he was just a bad little dog”, and comparing him to a child with Attention Deficit Disorder. A participant asked by the organization to keep the puppy for further training after one year decided to relinquish the puppy, stating that she and her husband were “drained in every way”, with lack of sleep, working full-time, and not being able to go out as usual. It got “depressing” and “overwhelming”, and the impact on the participant’s mental health was “shocking”. She was tired emotionally and physically, and it got “way too hard”. Another participant said that her physical and mental health had been damaged. She stated that she had shed many tears over the puppy, and felt angry with the organization and felt like a failure, “I felt for the puppy, that his life is not what it was destined to be. It was a struggle on all ends. I felt bad, and I felt like I was failing at that time, because I hadn't done enough teaching.”

Participants’ attachment to the puppy ranged from hatred, to no bond, to a close, reciprocal bond. For those who bonded, participants had to “mentally prepare” themselves for the puppy’s departure. For one participant, it was a “bit emotional” when she left, she was “our girl”, “we loved her a great deal”. For others, the departure was a relief.

A recurring theme throughout interviews was the lack of support from the organization. One participant felt that they lacked compassion for the puppy raisers, with their focus primarily on the dog. Their concerns and problems were not listened to or acted upon. Participants complained that they had not been given enough information about what to do and what to expect before receiving the puppy. It was also felt that the organization did not do anything to prepare participants for relinquishing the puppy or to check that they were coping emotionally when the puppy had left. Several participants were upset that they had not been told what had happened to the puppy since leaving their care, although one was not sure that she would want to know, if he had been euthanized. For several participants the notification that the puppy was to leave was short, with one who had been given four days notice feeling that this was not sufficient time to say “goodbye”—a planned celebration with the puppy and their family and friends could not take place. Participants did not feel that the organization had expressed gratitude for their work in any appropriate ways—such as a letter of thanks or phone calls. The puppy raisers of this litter arranged a birthday party for those who still had their animal at one year, and the support and friendship of others, through meeting via their role, was deemed rewarding and enjoyable.

Of the nine participants, one, as indicated above, had already taken a subsequent puppy, two had agreed to take puppies as short-term emergency boarders, two stated that they would consider being a puppy raiser when their work and family circumstances changed, and four participants stated they would definitely not do it again.

## 4. Conclusions

This study investigated the self-reported experiences of fostering a guide dog puppy, for a period of up to one year, for one litter of Labrador puppies and their nine raisers. Interviews were conducted at four time periods: before the puppy arrived into the household, one week after arrival, three months after arrival and at one year after arrival. The research question posed was “What is the impact of volunteering to raise a guide dog puppy on human physical, psychological and social health?” The answer to this question is that there were positive and negative impacts upon self-reported human health. Like those people with vision impairment who received a guide dog [5] the experience impacted life substantially, but for the participants in this study, the impacts were more negative than positive. In this qualitative study, as opposed to the survey study by Vail [14], the costs outweighed the benefits.

Prior to the arrival of the puppy, at Time 1, participants anticipated physical, psychological and social benefits from the puppy. At Time 2, participants were generally finding that these anticipated benefits had not materialised. At this point, stress was negatively impacting psychological health and psychological and physical health was affected by sleep deprivation and unanticipated and unwanted puppy behaviours. Social benefits had not yet been realised, as the puppy was not able to leave the house unvaccinated, and the puppy could not be left at any time for more than two hours. By Time 3, three puppies had already been returned. For those who still had a puppy, some physical, psychological and social benefits were noted, but overwhelmingly the effects of the puppy on these parameters were negative. At the final data collection point, Time 4, effects were again, primarily negative. Those who reported beneficial effects, interestingly, had the puppy in their care for shorter periods of time than those who had continued until the end of the 12-month period.

The mental health of volunteering has been the subject of several studies [12]. Whilst volunteering is usually considered as enhancing physical, psychological and social health, this study does not unequivocally support these assertions. Both the adequacy of training for the volunteer role and adequate ongoing support have been identified as factors that impact any benefits from volunteering [16]. It is clear from this study that in the eyes of the participants, the organization failed in their duty of care to them. Their experiences, and their health benefits, may have differed, if they had perceived that they had been adequately prepared, trained for and supported in their role.

Researchers have confirmed that dogs increase “social capital” [17,18] and increase sense of community [19]. Whilst participants gained friendships through meeting other puppy raisers, and some members of the community were friendly regarding the puppy, oftentimes social and community interchanges were a source of stress.

The puppies that came from the litter on which this research is based may not be typical of other guide dog puppies—they may have been a particularly boisterous or troublesome group. As a qualitative study there is no suggestion that the experiences of the participants in this research apply to all guide dog puppy educators. However, given that previous literature has not investigated the experiences associated with being a guide dog puppy raiser, this research makes an important contribution to existing knowledge. These data provide valuable information for guide dog schools, which rely on volunteers and their goodwill. Further research is needed to ensure that volunteers are being supported in their important roles in relation to the training of service animals. Future studies are needed to determine whether the puppies and raisers in this study are anomalies, or whether the challenges identified here are unusual or more typical. Qualitative and quantitative data on puppy progression through training to become a service dog, including retention and relinquishment with raisers is needed, to improve the experience for the puppies, the raisers, the vision impaired and blind recipients of the animals, and the organisations involved in breeding, training and allocation.
